# Genome Integrity in Dairy Cows Fed Black Soldier Fly Oil: An Integrated Sister Chromatid Exchange and Alkaline Comet In Vivo Assessment

**DOI:** 10.3390/genes17040404

**Published:** 2026-03-31

**Authors:** Alfredo Pauciullo, Giustino Gaspa, Viviana Genualdo, Cristina Rossetti, Angela Perucatti, Giulia Milanese, Martina Alessandra Gini, Flavia Caserta, Lara Rastello, Mathieu Gerbelle, Alessandro Galli, Laura Gasco, Manuela Renna

**Affiliations:** 1 Department of Agricultural, Forest and Food Sciences, University of Torino, Largo Paolo Braccini 2, 10095 Grugliasco, Italy; alfredo.pauciullo@unito.it (A.P.); fla.caserta@libero.it (F.C.); laura.gasco@unito.it (L.G.); 2 Institute for the Animal Production System in the Mediterranean Environment, National Research Council of Italy, Piazzale Enrico Fermi 1, 80055 Portici, Italy; viviana.genualdo@cnr.it (V.G.); cristina.rossetti@cnr.it (C.R.); angela.perucatti@cnr.it (A.P.); 3 Istituto Zooprofilattico Sperimentale del Piemonte, Liguria e Valle d’Aosta, Via Bologna 148, 10154 Torino, Italy; giulia.milanese@izsplv.it (G.M.); martina.gini@izsplv.it (M.A.G.); 4 Department of Veterinary Sciences, University of Torino, Largo Paolo Braccini 2, 10095 Grugliasco, Italy; lara.rastello@unito.it (L.R.); manuela.renna@unito.it (M.R.); 5 Institut Agricole Régional, Regione La Rochère 1/A, 11100 Aosta, Italy; m.gerbelle@iaraosta.it; 6 Ferrero Mangimi S.p.A., Via Fornace 15, 12060 Farigliano, Italy; alessandro.galli@ferreromangimi.it

**Keywords:** *Hermetia illucens* oil, ruminant, insect derived meal, genotoxicity, genome-damaging effects

## Abstract

Background/Objectives: Insect-derived lipids are emerging as circular-economy feed ingredients, but their implementation in ruminant diets requires robust safety assessment beyond productive endpoints. This study evaluated genome integrity in 26 lactating Valdostana Red Pied cows fed concentrates containing either hydrogenated palm fat (HPF; *n* = 13) or black soldier fly oil (*Hermetia illucens* oil, HIO; *n* = 13) for 50 days. Methods: Peripheral blood lymphocytes were analyzed using Sister Chromatid Exchanges (SCE), reflecting replication-associated chromosomal instability, and the alkaline Comet assay, quantifying primary DNA damage at the single-cell level (Tail DNA and Olive tail moment) at T0 (the day before the start of the two experimental diets), T1 (30 d) and T2 (50 d). Results: Baseline SCE frequencies were comparable between groups. Over time, SCE values decreased in both groups, but a significant reduction occurred only in HIO at day 50, with lower SCE frequency than HPF (5.73 ± 0.11 vs. 6.29 ± 0.13; *p* = 0.002). Comet tail DNA showed a significant time effect (T0 vs. T1: mean difference = 179,846.6; *p* < 0.001; T0 vs. T2: mean difference = 138,395.2; *p* = 0.012), with diet-dependent modulation. In fact, in HIO, tail DNA decreased from 387,886 ± 94,606 (T0) to 147,006 ± 30,592 (T1; *p* < 0.001), remained lower at day 50 (155,723 ± 29,357; *p* = 0.024), and was lower than HPF at both T1 (*p* = 0.006) and T2 (*p* = 0.009). Olive tail moment also decreased over time (T0 vs. T1: mean difference = 1.925 × 10^15^; *p* = 0.008; T0 vs. T2: mean difference = 1.676 × 10^15^; *p* = 0.025), and it differed between diets at day 50 in favor of HIO (5.99 × 10^15^ ± 5.45 × 10^14^ vs. 7.26 × 10^15^ ± 5.98 × 10^14^; *p* = 0.017). Conclusions: Overall, no evidence of genotoxicity was observed in cows fed HIO; conversely, the results support compatibility with genome stability and suggest a modest time-dependent improvement detectable mainly after prolonged supplementation.

## 1. Introduction

The growing need to rejoin animal production with environmental boundaries has accelerated interest in alternative feed ingredients that can simultaneously improve resource efficiency and reduce pressure on land and ecosystems. Within this context, the circular economy has emerged as an integrative approach aimed at sustaining the value of biological resources through cascading use, recycling of nutrients, and minimization of waste [1]. In livestock systems, circularity is increasingly translated into the search for feed ingredients able to “close loops” by converting low-value side streams into high-value nutrients, while maintaining safety for animals and, indirectly, for the human food chain [2].

Among the most promising solutions, insects have attracted attention as sustainable sources of proteins and lipids that can be produced by bioconversion of organic waste and by-products, with a potentially favorable land and water footprint [3]. In particular, the black soldier fly (*H. illucens*) can efficiently convert diverse substrates into valuable nutrients and bioactive compounds, contributing to waste reduction and nutrient recirculation [4]. At the same time, the nutritional and chemical composition of insect-derived products may vary with rearing conditions, substrate, and processing, an aspect that is central to both circular-economy claims and feed standardization [5]. This substrate-dependent variability has been documented in *H. illucens* production chains, where growth performance, waste reduction efficiency and chemical composition respond to the type of organic side stream used, including agro-industrial by-products [6]. From a ruminant-specific perspective, recent syntheses have also highlighted that regulatory frameworks and practical constraints differ between insect-derived oils and protein-rich insect meals, making insect lipids an especially relevant avenue to explore in ruminant nutrition [7,8]. Findings from other farmed species have further contributed to the rapid development of insect-feed research, as documented in early reviews [9].

While the sustainability benefits are clear, the inclusion of insect-derived ingredients in ruminant diets must be supported by rigorous safety evaluation. Beyond classical nutritional and productive endpoints, novel feed ingredients should be evaluated for potential unintended biological effects, including those linked to contaminants, oxidation products, or bioactive fractions that could influence cellular homeostasis. In this respect, genome integrity represents a high-level readout of organismal health. DNA and chromosomal lesions can arise from direct genotoxicants or indirectly through oxidative and inflammatory processes, and persistent genomic instability is biologically relevant because it may precede longer-term functional consequences.

Two complementary and historically grounded assays are widely used to investigate such outcomes: the Sister Chromatid Exchange (SCE) test and the Comet assay. The SCE test, established through differential staining of sister chromatids, provides a cytogenetic endpoint reflecting replication-associated events and the processing of DNA lesions during cell division [10,11]. Conversely, the alkaline Comet assay (single-cell gel electrophoresis) quantifies primary DNA damage at the single-cell level, mainly DNA strand breaks and alkali-labile sites, and is valued for its sensitivity to relatively subtle variations in baseline DNA integrity [12,13,14]. The combined use of cytogenetic (SCE) and molecular (Comet) tests can, therefore, improve interpretability, helping to distinguish between transient DNA strand breaks and replication-linked chromosomal instability, two layers that do not necessarily move in parallel.

In domestic animal research, SCE and Comet approaches have been used to assess genome safety in contexts directly relevant to feed innovation and environmental exposure. For SCE-based applications, for instance, Perucatti et al. [15] reported no cytogenetic toxicity in rabbit lymphocytes following feeding with verbascoside and/or lycopene; Iannuzzi et al. [16] applied the SCE test in river buffalo lymphocytes treated in vitro with furocoumarin extracts; and Genualdo et al. [17] combined SCE with redox homeostasis characterization in sheep flocks from Sardinian pasturelands. Importantly, Comet-based tests have also been applied in livestock systems, for example, to quantify lymphocyte DNA damage in pigs exposed to Fusarium mycotoxins and to evaluate the protective role of dietary vitamin E supplementation [18], as well as in bovine lymphocyte cultures exposed to pesticide formulations, often alongside cytogenetic tests, including SCE [19]. Together, these studies illustrate how validated biomarkers of genome integrity can complement nutritional and physiological readouts when introducing novel feed ingredients.

Within this conceptual background, the present study evaluates genome integrity in dairy cows fed diets with different lipid supplementation, namely hydrogenated palm fat (HPF) or *H. illucens* oil (HIO). By integrating the SCE test and the alkaline Comet assay in peripheral blood lymphocytes, this study aims to provide a biologically meaningful, safety-oriented assessment of whether an insect-derived lipid source, proposed within a circular-economy logic, induces in vivo detectable genotoxic effects or, conversely, is compatible with chromosomal stability and baseline DNA integrity.

## 2. Materials and Methods

### 2.1. Ethical Statement

The study protocol followed the provisions of the European Directive 2010/63/EU on the care and use of animals for scientific purposes and was approved by the Ethical Committee of the University of Turin (Protocol No. 0059643—30 January 2023).

### 2.2. Study Design, Animals and Diets

Twenty-six lactating Valdostana Red Pied cows belonging to the experimental farm of the Institut Agricole Régional located in Aosta (Italy) were used in this study. The animals were selected from a herd of 33 lactating cows and further divided randomly into two homogeneous groups of thirteen individuals each. The two groups were comparable for body weight (556 ± 49.5 kg), parity (3.0 ± 1.91), days in milk (68 ± 27.5), milk yield (22.0 ± 3.64 kg/cow/day) and milk composition (on average 34.5 ± 0.48 g/kg of fat, 31.4 ± 0.30 g/kg of protein, and 47.2 ± 0.15 g/kg of lactose).

Before the start of the experimental period, all the animals were maintained on the same feeding diet with an average forage-to-concentrate (F:C) ratio of 70:30 on a dry matter (DM) basis. The basal diet consisted of mixed hay, composed of 75% first-cut and 25% third-cut hay, and a concentrate comparable to those later employed in the trial but formulated without any lipid supplementation.

This study lasted for fifty days and included an initial two-week adaptation phase to the experimental diets. The forage component was identical to that offered before the trial and was supplied *ad libitum* to both groups. The pelleted concentrates provided to the animals were identical in composition (Table S1), except for the lipid source. Hydrogenated palm fat (HPF) obtained from Ferrero Mangimi SpA (Farigliano, Italy) was used for the control group (HPF group), whereas *H. illucens* oil (HIO) supplied by Mutatec (Cavaillon, France) was included for the treatment group (HIO group). Lipid inclusion was set at 3% of the concentrate as fed. Further details on animal diet and fatty acid composition of both HPF and HIO are reported by Rastello et al. [20].

### 2.3. Cell Culture and Banding Techniques

Peripheral blood samples were collected from each cow by using sterile Vacutainer tubes (Becton Dickinson Italia S.p.A., Milano, Italy) containing sodium heparin. According to the protocol as previously published [15,21], two different cultures were set up per sample: normal cultures (without the addition of any analog base), used for the karyotyping by G bands by Trypsin and Giemsa (GTG-banding), and cultures treated for the late incorporation of 5-bromo-2’-deoxyuridine (5-BrdU) (Sigma-Aldrich; St. Louis, MO, USA) for the SCE test. For the SCE test, three time points were considered: zero time (T0—no HPF or HIO inclusion in both animal groups), 30 days (T1), and 50 days (T2) after the start of the experimental feeding.

### 2.4. Comet Assay Test

Fresh blood samples were collected in K_2_EDTA tubes (Becton Dickinson Italia S.p.A., Milano, Italy) from each cow. After centrifugation, the buffy coat was recovered and treated with Histopaque-1077 (Sigma-Aldrich, Darmstadt, Germany) to remove the erythrocyte contamination and get lymphocytes ready for the Comet assay.

Comet assays were performed under alkaline conditions as described by Singh et al. [13]. Briefly, 1 × 10^5^ lymphocyte cells/mL, assessed by Bürker chamber, were combined with molten LM Agarose (at 37 °C) (Sigma-Aldrich; St. Louis, MO, USA) at a 1:10 (*v*/*v*) ratio. Slides were prepared and chilled at 4 °C for 30 min. Afterwards, cells were lysed at 4 °C in a lysis solution (2.5 M NaCl, 100 mM EDTA, pH 10, 10 mM Tris Base, 1% sodium lauryl sarcosinate, and 1% Triton x-100) supplemented with 10% DMSO to neutralize the Reactive Oxygen Species (ROS) potentially generated by heme groups from residual red cells. This avoided DNA oxidative artifacts. Alkaline treatment (300 mM NaOH, 1 mM EDTA, and pH = 13) was then performed at room temperature for 45 min in the dark before the electrophoresis, which was still achieved in alkaline solution at 4 °C for 30 min at 300 mA. A washing step in deionized water followed by a dehydration step in 70% ethanol for 5 min was accomplished before air-drying slides. Staining was performed with SYBR^®^ Green I (Sigma-Aldrich, Darmstadt, Germany) for 30 min at room temperature in the dark. The excess of staining solution was briefly removed in deionized water, and the slides were completely dried at 37 °C before microscope observation.

### 2.5. Microscopic Analysis

At least 30 metaphases were examined from slides treated by GTG-banding techniques, and only the complete metaphases (2n = 60, XX) were analyzed for the karyotypes, arranged according to the cattle standard karyotype [22].

The 5-BrdU-treated cultures for the SCE test were stained using a modified Fluorescence Plus Giemsa technique [11]. To visualize SCE, slides were stained with Hoechst 33,258 (25 mg/mL) (Sigma-Aldrich; St. Louis, MO, USA) for 20 min at room temperature, washed with distilled water and exposed to UV light for about 30 min. Afterwards, slides were rinsed with distilled water and stained for 10 min with acridine orange (0.01% in buffer phosphate), washed again with distilled water, and mounted in P-buffer. On average, at least 35 complete metaphases for each animal and for each time point were analyzed. SCEs were manually counted by the same operators for all analyzed experimental times (T0, T1, T2).

All metaphases were observed under the Leica DM2000 (Leica, Wetzlar, Germany) bright field and fluorescence microscope equipped with 100× oil immersion lenses, FITC-specific filters and a camera. Each image was processed using dedicated software (CytoVision DX platform by Leica).

The comet slides were observed at 20× magnification with a Zeiss Axioscope 5 epifluorescence microscope (Zeiss, Oberkochen, Germany) equipped with a FITC-specific filter and provided with the Axiocam 208 color camera (Zeiss, Oberkochen, Germany) and the Zen image-analysis software ver. 3.13. Two slides per sample were analyzed, and at least 50 individual cells per animal were randomly chosen and screened per subject (at least 25 cells from each slide). OpenComet software ver. 1.3 was used for scoring the comet characteristics according to Gyori et al. [23]. Comets occurring in clusters or at the margins of the image were excluded from the analysis. From the subset of valid comets, additional validation steps were performed to classify comets according to their outline characteristics as follows: red outline and number (normal comets); yellow outline and number (outlier comets); and gray outline (removed shape). Each image was manually checked, and incorrectly found comets were removed.

### 2.6. Statistical Analyses

SCE and Comet data distribution within each group was tested for normality using the Shapiro and Wilk test [24]. The individual animal (cow) was considered the experimental unit, while metaphases and comet assay observations were treated as repeated measurements within each individual. Data were summarized prior to statistical analysis as mean values per animal for SCE and as median values per animal for the Comet assay. After verification of normality, data were analyzed by two-way ANOVA (JASP, ver. 9.2) using total SCEs (or Tail DNA or Olive tail moment) as the dependent variable, whereas time and diet were used as fixed factors, including their interaction (time × diet). Post hoc pairwise comparisons were performed using the Bonferroni correction. *p*-values ≤ 0.05 were considered significant, whereas 0.05 < *p*-values ≤ 0.10 were interpreted as a trend toward significance.

## 3. Results

### 3.1. Sister Chromatid Exchange Test

**Figure 1:**
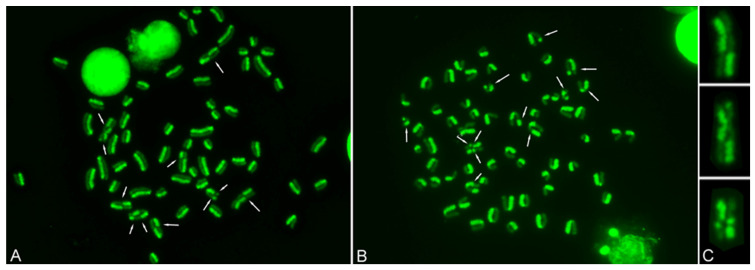
Sister Chromatid Exchange (SCE) test in the Valdostana Red Pied cows. Metaphases stained with acridine orange (green) showing several SCEs (arrows) (**A**,**B**). Detail of the X chromosome showing, from top to bottom, single, double, and triple SCEs (**C**).

A total of 2882 metaphases were elaborated, and a total of 18,352 SCEs were counted, dividing them into single (16,484), double (889), and triple (30) SCEs. Figure 1 shows examples of metaphases with single, double, and triple SCEs.

SCE-mean values tended to decrease within the same group, starting from T0 to T2 (50 days) (Table 1), but the differences were statistically significant only for the T0 vs. T2 (6.72 ± 0.14 vs. 5.73 ± 0.11) in the HIO group (*p* < 0.001) (Table 1).

Post hoc comparison analysis showed that the first 30 days were sufficient in establishing significant differences in the total SCE number (Table 2), and that diet had a significant effect on SCEs (Table 3).

Furthermore, statistical analysis showed no significant differences in SCE frequency between the HPF and HIO groups at T0 (6.84 ± 0.15 vs. 6.72 ± 0.14; *p* = 0.989) and T1 (6.30 ± 0.13 vs. 6.27 ± 0.13; *p* = 1.000). However, after 50 days (T2), a significant reduction in SCE frequency was observed in the HIO group compared to the HPF one (5.73 ± 0.11 vs. 6.29 ± 0.13; *p* = 0.002) (Table 4).

### 3.2. Comet Assay

**Figure 2:**
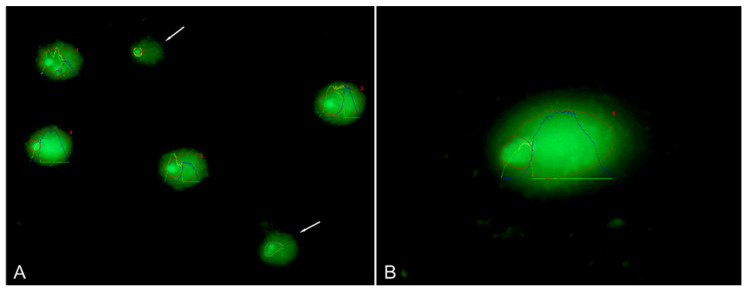
Comet assay test in the Valdostana Red Pied cows. Comets stained with SYBR^®^ Green I, observed at 20× magnification and analyzed using OpenComet software ver. 1.3 (red: normal comet; green profile: comet head; yellow profile: comet tail; and blue profile: comet head and tail). Incorrectly recognized comet outlines were manually excluded from the analysis. Arrows (from top to bottom) indicate a yellow outlier and a gray removed shape, respectively (**A**). Detail of a normal comet analyzed at 100× magnification (**B**). Red numbers indicate the identified comets in an analyzed microscopic image (**A**,**B**).

A total of 4063 lymphocyte nuclei were analyzed by the Comet assay to evaluate DNA damage, expressed as Tail DNA and Olive tail moment (Figure 2). Descriptive statistics for both parameters are reported in Table 1.

For Tail DNA, a significant effect of time was observed irrespective of diet (Table 2). In particular, Tail DNA values significantly decreased from T0 to T1 (mean difference = 179,846.6; *p* < 0.001) and from T0 to T2 (mean difference = 138,395.2; *p* = 0.012), whereas no significant differences were detected between T1 and T2. No overall significant effect of diet was found when comparing HPF and HIO groups, although a trend toward significance has been observed (Table 3; *p* = 0.081).

The post hoc analysis of the time × diet interaction highlighted a diet-dependent modulation of Tail DNA over time (Table 4). In the HIO group, Tail DNA values showed a marked and significant reduction at T1 and T2 compared to T0 (*p* < 0.001 and *p* = 0.019, respectively), whereas no significant differences were observed among time points within the HPF group. Furthermore, at both T1 and T2, Tail DNA values were significantly lower in the HIO group compared to the HFP one (*p* = 0.005 and *p* = 0.008, respectively).

A similar temporal pattern was observed for the Olive tail moment. The main effect of time was significant (Table 2), with a decrease from T0 to T1 (mean difference = 1.93 × 10^15^; *p* = 0.008) and from T0 to T2 (mean difference = 1.68 × 10^15^; *p* = 0.025), while no significant differences were detected between T1 and T2. The overall effect of diet on the Olive tail moment was not significant (Table 3; *p* = 0.140).

The time × diet interaction analysis revealed that, within the HIO group, the Olive tail moment significantly decreased at T2 compared to T0 (*p* = 0.017), whereas no significant temporal variations were detected within the HPF group (Table 4).

## 4. Discussion

In the present study, genome integrity in dairy cows was evaluated by integrating SCE analysis with the alkaline Comet assay, two complementary approaches that capture distinct layers of genetic damage. SCEs, classically scored after differential staining of sister chromatids, provide a cytogenetic readout of replication-associated chromosomal instability and the processing of DNA lesions during cell division [10]. Conversely, the alkaline Comet assay is widely used to quantify primary DNA damage at the single-cell level (mainly DNA strand breaks and alkali-labile sites), and it is particularly valuable for detecting relatively subtle and transient changes in DNA integrity in vivo [12,14,25,26]. The combined use of these endpoints is, therefore, useful to describe whether a dietary change affects transient DNA lesions (Comet) and/or longer-term chromosomal stability (SCE), which do not necessarily change in parallel.

At baseline (T0), the two dietary groups (HPF and HIO) showed comparable SCE frequencies (6.84 ± 0.15 vs. 6.72 ± 0.14), indicating that the groups started from a similar level of chromosomal stability. This is important because spontaneous SCE rates in cattle can vary with multiple factors (including genetic background and experimental conditions), and, therefore, baseline comparability strengthens the interpretation of subsequent diet-related divergence.

Across the experimental period, SCE-mean values tended to decrease in both groups. However, the reduction was significant only in the cows receiving *H. illucens* oil after 50 days, with a significant separation from HPF-fed cows at T2. Specifically, at T2, the HIO group displayed a significantly lower SCE frequency than the HPF group. This is consistent with a time-dependent modulation rather than an immediate response, which is biologically plausible, given that SCEs are expressed as outcomes of replication and recombination events occurring across cell cycles [10]. Importantly, the SCE values observed across the study (≈5.7–6.8 SCE/cell across time and diet) fall within the range previously reported in bovine lymphocytes, although baseline levels can vary among studies and breeds depending on experimental conditions and scoring procedures [27,28]. In the global diet contrast, diet also showed a significant effect (*p* = 0.034). In this context, the emergence of a statistically supported difference at the end of the trial suggests that the insect-derived lipid source may influence chromosomal stability only after sufficient exposure, rather than reflecting a pre-existing divergence between groups.

The observed pattern also aligns with how SCE has been used in other livestock contexts to discriminate between conditions associated with genomic stress versus those compatible with genome stability. For example, sheep flocks grazing in environmentally impacted areas showed evidence of increased chromosomal damage (SCE), together with altered redox homeostasis [17], supporting the concept that SCE is responsive to systemic oxidative/inflammatory contexts in farm animals. In a different setting, rabbit lymphocytes from animals fed verbascoside and/or lycopene did not show cytogenetic toxicity [15]. This evidence is further supported by recent findings in rabbits fed mulberry leaf meal, where no increase in SCE frequency was detected compared to controls, confirming the absence of diet-related chromosomal instability and reinforcing the genomic safety profile of this alternative feed ingredient [21]. Likewise, river buffalo lymphocytes exposed in vitro to furocoumarin extracts were evaluated with SCE to screen mutagenic potential under controlled conditions [16]. Taken together, these and other examples in the literature support the use of SCE as a method to verify a “safety” interpretation for bioactive dietary components.

Comet assays provided additional, partly independent information. Tail DNA and Olive tail moment, two commonly reported descriptors recommended in the literature, were selected as primary information to capture both the extent of migrated DNA and a combined measure incorporating migration and DNA distribution [12,14,25,26].

In contrast to SCE, the Comet assay did not show a significant overall diet effect for either Tail DNA (*p* = 0.081) or Olive tail moment (*p* = 0.140). Conversely, time had an influence on both Comet data. Tail DNA decreased from T0 to T1 (*p* < 0.001) and remained lower at T2 compared with baseline (T0 vs. T2). Olive moment similarly decreased from T0 to T1 (*p* = 0.007) and from T0 to T2 (*p* = 0.023). The time × diet comparisons indicated that the HIO group exhibited a more consistent reduction over time, and lower values than controls at later time points for Tail DNA (T1 and T2) and for Olive tail moment at T2. This pattern may reflect the intrinsic characteristics of the alkaline Comet assay in vivo. The method is highly sensitive, but also prone to substantial biological and technical variability, so diet-related differences can be more detectable at specific time points than as a single pooled “main effect” [12,14,25,26]. Moreover, because strand breaks and alkali-labile sites can be efficiently repaired, Comet data may fluctuate with short-term physiological changes and may not necessarily move in parallel to chromosomal indicators such as SCEs, which integrate events linked to replication and lesion processing across time [12,25,26].

Considering both biomarkers together, the results do not indicate any genotoxic effect of *H. illucens* oil; instead, they support a scenario in which the dietary utilization of HIO instead of HPF is compatible with genome stability and may be associated with modest improvements detectable primarily after prolonged supplementation. The present work was not designed to dissect pathways, but the time dependence observed for both SCE and Comet data is compatible with gradual metabolic adaptation to the dietary lipid source. In this regard, *H. illucens*-derived lipids are increasingly explored as sustainable alternatives in animal nutrition [29], and their fatty acid profile can be influenced by rearing substrate and processing, potentially affecting downstream physiological responses [30,31,32], which should be further evaluated.

In dairy cows specifically, the replacement of HPF with HIO has been investigated in relation to environmental sustainability, as well as digestive parameters, oxidative stress, and production outcomes, supporting the relevance of examining genome integrity endpoints alongside more classical performance and metabolic readouts [20]. Additionally, feeding *H. illucens* fat to dairy cows has been reported without negative effects on health and production traits, which is consistent with the general safety-oriented interpretation of the present cytogenetic results [33,34]. While these nutritional studies do not automatically imply genoprotective effects, they do support the biological plausibility that changes in systemic oxidative balance and metabolism could occur under such dietary substitutions, an important upstream determinant of DNA damage processes [20].

Reviews on *H. illucens* lipids also highlight variability in composition and the possible presence of minor bioactive fractions, raising the hypothesis that insect-derived fats could influence redox balance and inflammatory tone depending on context [32]. Experimental comparisons of extracted oils from different sources have further reported differences in antioxidant-related properties and bioactive compounds, which provides an additional rationale, while still indirect, for evaluating genome integrity data alongside traditional production traits [35].

A relevant consideration is that the alkaline Comet assay primarily captures strand breaks and alkali-labile sites, while oxidative base lesions are more specifically detected using enzyme-modified versions of the assay (e.g., Fpg or EndoIII), which can increase sensitivity to oxidative DNA damage [12,14]. Given that nutritional change may alter oxidative status without producing large shifts in basal strand breaks, enzyme-modified Comet protocols combined with systemic oxidative stress markers could strengthen mechanistic inference and clarify whether the observed time trends are driven by adaptation, altered DNA repair activity, or broader metabolic effects [12,14,36].

## 5. Conclusions

Taken together, our results indicate that dietary *H. illucens* oil did not induce detectable genotoxic effects by either SCE or Comet assays. Instead, SCE findings suggest a potential beneficial effect of HIO on chromosomal stability over time, whereas the Comet assay mainly reflected general temporal dynamics rather than diet-specific responses. This integrated interpretation is consistent with the idea that, if present, benefits to genome integrity may be more evident in replication-associated markers such as SCEs than in basal levels of DNA strand breaks measured under alkaline Comet assay conditions.

## Figures and Tables

**Table 1:** Descriptive statistics of Sister Chromatid Exchange (SCE) and Comet assay (Tail DNA and Olive tail moment) in Valdostana Red Pied cows fed diets containing hydrogenated palm fat (HPF) or *H. illucens* oil (HIO).

Test	Animal Diet	Time											
		T0				T1				T2			
		N. Cells	Mean	SD	SEM	N. Cells	Mean	SD	SEM	N. Cells	Mean	SD	SEM
SCEs	HPF	495	6.84	3.48	0.15	472	6.30	2.75	0.13	457	6.29	2.73	0.13
	HIO	489	6.72	3.30	0.14	490	6.27	2.85	0.13	479	5.73	2.60	0.11
Comet													
T DNA	HPF	491	219,315	132,946	44,315	776	100,501	40,410	11,207	751	174,687	109,059	30,247
	HIO	523	387,886	299,173	94,606	769	147,006	110,301	30,592	753	155,723	105,851	29,357
OTM	HPF	491	9.01 × 10^15^	1.49 × 10^15^	4.98 × 10^14^	776	6.13 × 10^15^	1.76 × 10^15^	4.89 × 10^14^	751	7.26 × 10^15^	2.16 × 10^15^	5.98 × 10^14^
	HIO	523	7.59 × 10^15^	2.24 × 10^15^	7.08 × 10^14^	769	6.62 × 10^15^	1.95 × 10^15^	5.41 × 10^14^	753	5.99 × 10^15^	1.96 × 10^15^	5.45 × 10^14^

Abbreviations: SD, standard deviation; SEM, standard error of the mean; T DNA, Tail DNA; OTM, Olive Tail Moment; T0 (day zero); T1 (30 days); T2 (50 days).

**Table 2:** Post hoc comparisons for the main effect of Time on SCEs and comet assay parameters.

			Mean Difference	SE	t	P_Bonf_
SCEs	T0	T1	0.497	0.135	3.685	<0.001
	T0	T2	0.768	0.136	5.654	<0.001
	T1	T2	0.271	0.137	1.983	0.142
Tail DNA	T0	T1	179,846.585	46,399.921	3.876	<0.001
	T0	T2	138,395.181	46,399.921	2.983	0.012
	T1	T2	−41,451.404	42,604.410	−0.973	1.000
Olive Tail Moment	T0	T1	1.925 × 10^15^	6.165 × 10^14^	3.122	0.008
	T0	T2	1.676 × 10^15^	6.165 × 10^14^	2.718	0.025
	T1	T2	−2.490 × 10^14^	5.661 × 10^14^	−0.440	1.000

**Table 3:** Difference contrast for the main effect of Diet (hydrogenated palm fat versus *H. illucens* oil) on SCEs and Comet assay parameters.

Test	Comparison	Estimate	SE	df	t	*p*
SCEs	HPF vs. HIO	0.235	0.111	2876	2.118	0.034
Tail DNA	HPF vs. HIO	−65,370.890	36,881.314	65	1.772	0.081
Olive Tail Moment	HPF vs. HIO	−7.319 × 10^14^	4.901 × 10^14^	65	−1.494	0.140

**Table 4:** Post hoc comparisons for the time × diet interaction on SCEs and Comet assay parameters. Above the diagonal: the mean difference between the variables, and the standard error in brackets. Below the diagonal: the *p*-values corrected by Bonferroni.

SCE	Diet		HFP			HIO		
		Time	T0	T1	T2	T0	T1	T2
	HPF	T0	-	0.544 (0.191)	0.547 (0.193)	−0.118 (0.190)	−0.569 (0.190)	−1.107 (0.191)
		T1	0.068	-	0.003 (0.195)	0.425 (0.192)	−0.025 (0.192)	−0.564 (0.193)
		T2	0.069	1.000	-	0.428 (0.194)	−0.022 (0.193)	−0.561 (0.194)
	HIO	T0	1.000	0.403	0.404	-	0.450 (0.190)	0.989 (0.191)
		T1	0.041 *	1.000	1.000	0.268	-	0.539 (0.191)
		T2	<0.001 **	0.052	0.002 **	<0.001 **	0.073	-
T DNA	Diet		HFP			HIO		
		Time	T0	T1	T2	T0	T1	T2
	HPF	T0	-	118,813 (66,610)	44,627 (66,610)	−16,857 (70,580)	72,308 (66,610)	63,591 (66,610)
		T1	1.000	-	−74,186 (60,251)	−287,384 (64,612)	−46,505 (60,251)	−55,222(60,251)
		T2	1.000	1.000	-	−213,198 (64,612)	−27,680 (60,251)	18,964 (60,251)
	HIO	T0	0.298	<0.001 **	0.024 *	-	240,879 (64,612)	232,162 (64,612)
		T1	1.000	1.000	1.000	0.006 **	-	−8716 (60,251)
		T2	1.000	1.000	1.000	0.009 **	1.000	-
OTM	Diet		HFP			HIO		
		Time	T0	T1	T2	T0	T1	T2
	HPF	T0	-	2.88 × 10^15^ (8.85 × 10^14^)	1.75 × 10^15^ (8.85 × 10^14^)	1.42 × 10^15^ (9.38 × 10^14^)	2.39 × 10^15^ (8.85 × 10^14^)	3.02 × 10^15^ (8.85 × 10^14^)
		T1	0.027 *	-	−1.13 × 10^15^ (8.01 × 10^14^)	1.46 × 10^15^ (8.59 × 10^14^)	−4.90 × 10^15^ (8.01 × 10^14^)	1.39 × 10^15^ (8.01 × 10^14^)
		T2	0.777	1.000	-	3.33 × 10^15^ (8.59 × 10^14^)	−6.36 × 10^15^ (8.01 × 10^14^)	1.27 × 10^15^ (8.01 × 10^14^)
	HIO	T0	1.000	1.000	1.000	-	9.70 × 10^15^ (8.59 × 10^14^)	1.60 × 10^15^ (8.59 × 10^14^)
		T1	0.132	1.000	1.000	1.000	-	6.29 × 10^15^ (8.01 × 10^14^)
		T2	0.017 *	1.000	1.000	1.000	1.000	-

Abbreviations: T DNA, Tail DNA; OTM, Olive Tail Moment; T0 (day zero), T1 (day 30), T2 (day 50). * *p* ≤ 0.05; ** *p* ≤ 0.01.

## Data Availability

The data presented in this study are available on request from the corresponding author.

## Supplementary Materials

The following supporting information can be downloaded at: https://www.mdpi.com/article/10.3390/genes17040404/s1, Table S1: Ingredient composition (g/100 g DM) of the two experimental concentrates containing hydrogenated palm fat (HPF) or *H. illucens* oil (HIO) [20].

## Abbreviations

The following abbreviations are used in this manuscript:

SCE	Sister Chromatid Exchange
HIO	*Hermetia illucens* Oil
HPF	Hydrogenated Palm Fat
T DNA	Tail DNA
OTM	Olive Tail Moment
DM	Dry Matter
GTG-banding	G bands by Trypsin and Giemsa
BrdU	5-bromodeoxyurdine
EDTA	Ethylenediaminetetraacetic Acid
DMSO	Dimethyl Sulfoxide
FITC	Fluorescein Isothiocyanate
