# Supplementary material for: Genome Integrity in Dairy Cows Fed Black Soldier Fly Oil: An Integrated Sister Chromatid Exchange and Alkaline Comet In Vivo Assessment
Source: Genes (Basel). 2026 Mar 31;17(4):404. doi: 10.3390/genes17040404 (PMC13116937; doi:10.3390/genes17040404)
Supplement: Supplementary file 1 [file genes-17-00404-s001.zip › genes-4201406-supplementary.pdf]

Table S1. Ingredient composition (g/100 g DM) of the two experimental concentrates containing hydrogenated palm fat (HPF) or *H. illucens* oil (HIO) [20].

Ingredients	Dietary treatment	
	HPF	HIO
Corn meal	29.5	29.5
Dehulled sunflower meal (36% CP)	17.0	17.0
Wheat bran	16.0	16.0
Wheat middlings	10.6	10.6
Barley meal	7.8	7.8
Soybean meal (44% CP)	5.9	5.9
Palm hydrogenated fat	3.4	-
Hermetia illucens oil	-	3.4
Pitted carobs	3.2	3.2
Calcium carbonate ($CaCO_3$)	2.3	2.3
Cane molasses	1.2	1.2
Sodium chloride (NaCl)	1.1	1.1
Dolomite ($CaMg(CO_3)_2$)	1.1	1.1
Sodium bicarbonate	0.6	0.6
Vitamin mixture[a]	0.3	0.3

1. *CP* Crude protein
2. [a]Containing per 100 g of product: Vitamin A, 20,000 IU; Vitamin D_3, 1,600 IU; Vitamin E, 40 mg.
3. For further details about the experimental diets and rearing conditions, please refer to Rastello et al. [20].
